# Unlocking the Health Secrets of Onions: Investigating the Phytochemical Power and Beneficial Properties of Different Varieties and Their Parts

**DOI:** 10.3390/molecules30081758

**Published:** 2025-04-14

**Authors:** Adele Muscolo, Angela Maffia, Federica Marra, Santo Battaglia, Mariateresa Oliva, Carmelo Mallamaci, Mariateresa Russo

**Affiliations:** Department of Agraria, Mediterranea University, Feo di Vito, 89122 Reggio Calabria, Italy; angela.maffia@unirc.it (A.M.); federica.marra@unirc.it (F.M.); santo.battaglia@unirc.it (S.B.); mariateresa.oliva@unirc.it (M.O.); carmelo.mallamaci@unirc.it (C.M.); mariateresa.russo@unirc.it (M.R.)

**Keywords:** onion bioactive compounds, phenolic compounds, antioxidant activity, Tropea red onion, functional food, flavonoids, drying technology

## Abstract

Onions (*Allium cepa* L.) are widely consumed worldwide and are recognized for their high content of bioactive compounds with potential health benefits. This study investigates the nutritional and phytochemical properties of three onion varieties—Tropea red onion, red onion, and yellow onion—analyzed in their whole form as well as in their peel and pulp. An innovative drying system was employed to assess its impact on the retention of bioactive compounds. The results highlight significant differences in nutrient composition among varieties and onion parts. The peel exhibited the highest concentrations of proteins, phenolic compounds, flavonoids, and antioxidants, followed by the whole onion and pulp. Tropea red onion stood out for its superior antioxidant capacity, vitamin C content, and phenolic acid levels, reinforcing its potential for functional food applications. This study also revealed that mineral content, particularly calcium, potassium, and sulfates, varied across onion varieties, influencing their nutritional and health-promoting properties. These findings support the valorization of onion byproducts for their bioactive potential and sustainability in the food industry. The data emphasize the need for further research on innovative processing techniques that enhance the bioavailability and effectiveness of onion-derived health-promoting compounds.

## 1. Introduction

Onion (*Allium cepa* L.) is one of the most widely consumed vegetables worldwide, with its origins tracing back to Central Asia [1]. It is among the oldest cultivated crops, with evidence of its cultivation spanning over 4000 years. Onions are rich in bioactive compounds, which contribute to various health benefits, including anti-inflammatory, antioxidant, anti-obesity, antidiabetic, anticancer, antiallergic, cardiovascular-protective, neuroprotective, respiratory-protective, and bacteriostatic effects [2,3]. These properties make onions valuable not only in culinary uses but also in medicinal applications. The synthesis of bioactive compounds in onions is influenced by several factors, such as cultivar type and environmental conditions where the onions grow. Variations in the geographical area, climate, soil composition, and cultivation practices can lead to significant differences in the levels of these beneficial compounds across onion varieties [4]. The main differences in biocompound content between onion varieties lie in the levels of sulfur compounds, flavonoids, phenolic compounds, and anthocyanins, with red onions generally having the highest concentrations of antioxidant-rich compounds [5]. These differences contribute to the distinct health benefits and culinary characteristics of each variety. The primary differences in terms of biocompounds are related to specific allyl propyl disulfide and S-allyl cysteine, sulfur compounds responsible for the characteristic odor and taste of onions, with potential health benefits, including anti-inflammatory and anticancer properties [6,7]. Varieties like red onions generally have higher concentrations of sulfur compounds compared to yellow or white onions [8]. They also contain the highest levels of quercetin, primarily in their outer layers [9]. Yellow onions, while also rich in flavonoids, typically have lower concentrations than red varieties [4]. Phenolic compounds, including ferulic and caffeic acids, exhibit antioxidant and anti-inflammatory properties, with red onions containing higher levels than yellow or white onions [10]. Anthocyanins, responsible for antioxidant and anti-inflammatory effects, are found mainly in red and purple onions, contributing to their vibrant color [11].

Onions are also a good source of vitamins C and B6, along with minerals such as potassium and manganese. Although present in all varieties, their concentrations vary depending on the onion type and growing conditions [12]. Tropea red onions, a specific variety from Calabria, Italy, share similarities with regular red onions but have distinct characteristics that enhance their nutritional value and health benefits. They are particularly rich in anthocyanins, flavonoids (especially quercetin), and phenolic compounds, offering superior antioxidant properties and potential cardiovascular benefits [13]. Their higher sugar content gives them a sweeter taste, while a lower sulfur concentration results in a milder flavor. Additionally, they contain slightly higher mineral levels, further enhancing their nutritional profile [13].

With the increasing demand for longer-shelf-life products, the market for processed onions, such as dehydrated, frozen, and canned forms, has expanded significantly. Innovations in preservation techniques, including vacuum drying, freeze-drying, and advanced freezing methods, have improved the retention of antioxidants, vitamins, and other bioactive compounds [14]. While most research has focused on the chemical composition and health benefits of onions, fewer studies have examined how bioactive compounds are distributed across different parts of the onion. This study analyzes the composition of red, Tropea red, and yellow onions, specifically their peel, pulp, and whole form, after treatment with an innovative dry concentration system by Gioia Succhi Food Industry. The goal is to compare cultivars, assess differences in chemical composition across onion parts, and evaluate the impact of the drying method. Additionally, this study explores ways to utilize onions unsuitable for the food market in disease prevention and metabolic disorder management.

## 2. Results

**Figure 1:**
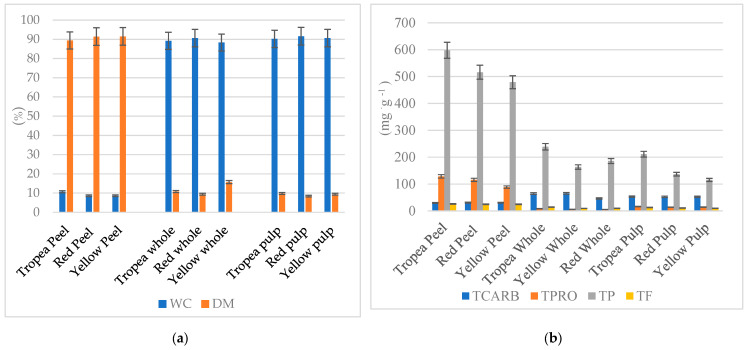
(**a**,**b**): Water content (WC %) and dry matter (DM %) (**a**) and total carbohydrates (TCARB, mg·g^−1^ dw), total protein (TPRO, mg BSA·g^−1^ dw), total phenols (TP, mg GAE·g^−1^), and total flavonoids (TF, mg QE·g^−1^) (**b**) in Tropea red onion (whole, peel, and pulp), red onion (whole, peel, and pulp), and yellow onion (whole, peel, and pulp).

The three onion varieties did not show significant differences in water content and dry matter. However, notable variations were observed in protein and carbohydrate content, as well as their distribution across different onion parts. The peel consistently had the highest nutrient concentration across all varieties, followed by the pulp, while whole onions had the lowest levels. This lower nutrient content in whole onions was largely attributed to the high-water content in the pulp, identified as the most water-rich part (Figure 1a).

Regarding macronutrient composition, protein content was significantly higher in the peel, while carbohydrates were more concentrated in the pulp. Among the three varieties, Tropea exhibited the highest protein levels across all onion parts. The red onion varieties, including Tropea, contained a greater total carbohydrate content than the yellow onion. Analysis of phenolic compounds revealed that total phenols were predominantly found in the peel, with Tropea having the highest levels, followed by whole onions and then the pulp. The yellow onion had the lowest phenol content among the varieties (Figure 1b).

Flavonoid distribution followed a similar pattern to phenols, with the highest concentrations in the peel, followed by whole onions and the pulp. However, flavonoid levels were approximately 20 times lower than total phenols (Figure 1b).

**Figure 2:**
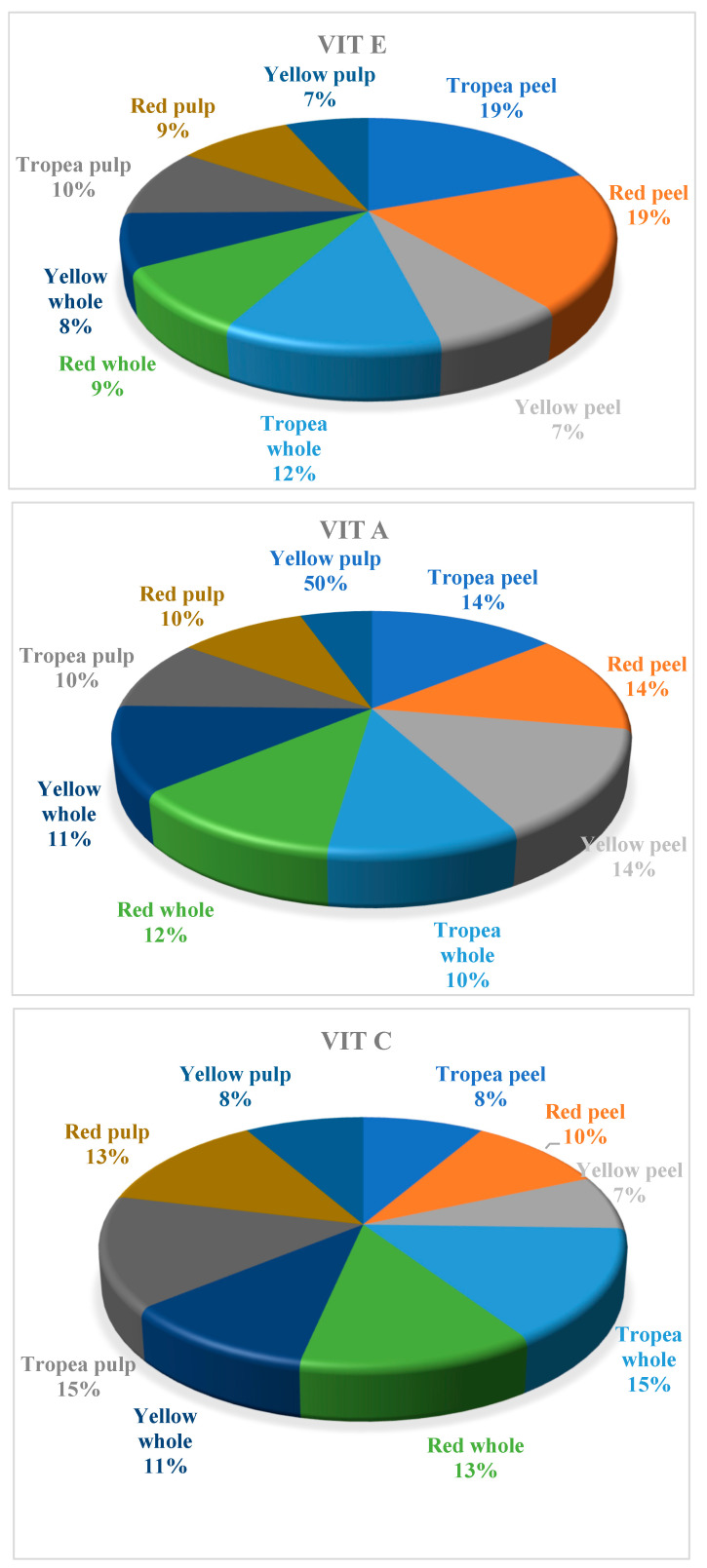
Distribution (%) of vitamin A (VIT A, µg RE*100 g^−1^ DM), vitamin C (VIT C, mg ASC*100 g^−1^ DM), and vitamin E (VIT E, mg α-tocopherol*100 g^−1^ DM) in Tropea red onion (whole, peel, and pulp), red onion (whole, peel, and pulp), and yellow onion (whole, peel, and pulp). Values are expressed as percentages representing the proportion of the total content of each vitamin (A, C, and E) distributed across the different onion samples.

**Figure 3:**
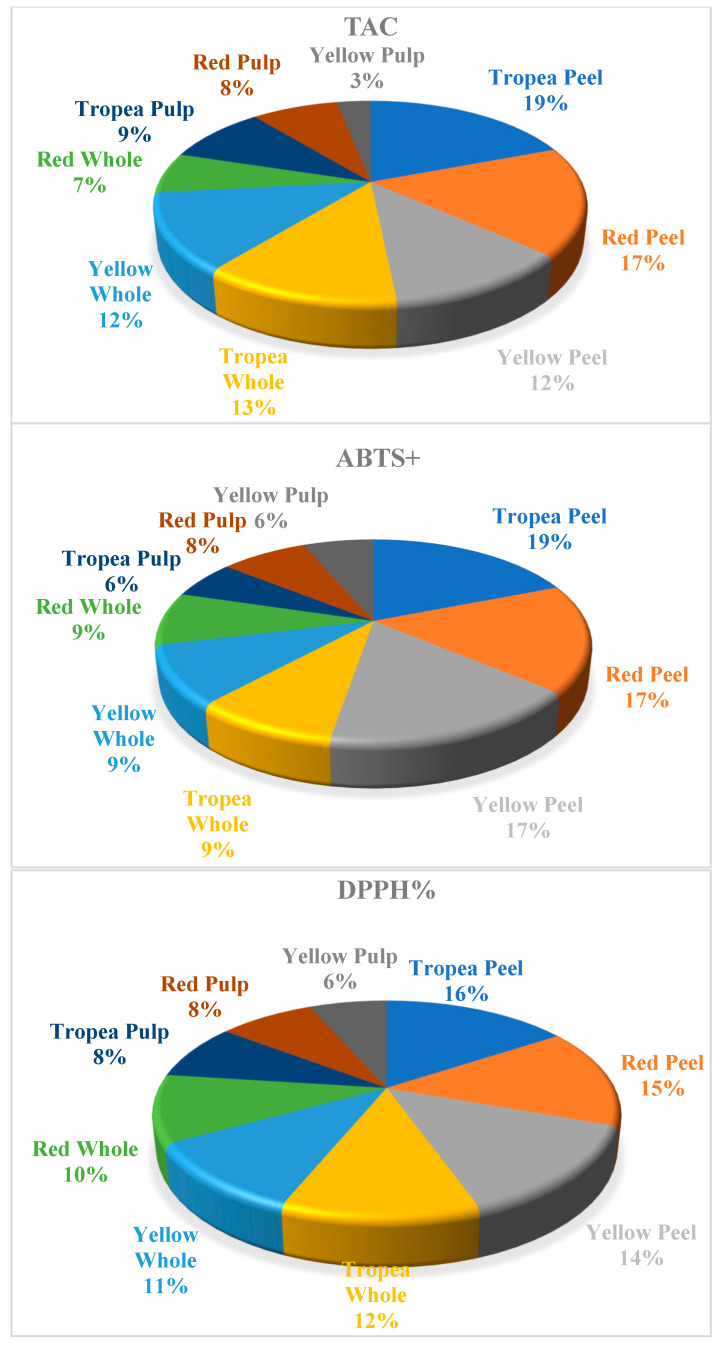
Antioxidant activity of 2,2-diphenyl-1-picryl-hydrazyl-hydrate (DPPH, % inhibition), 2,2-diphenyl-1-picryl-hydrazyl-hydrate (DPPH, µmol TE*g^−1^), 2,2′-azino-bis-3-ethylbenzothiazoline-6-sulfonic acid (Abts+ % inhibition), and total antioxidant capacity (TAC, mg α-tocoferolo*100 g^−1^ DM) in Tropea red onion (whole, peel, and pulp), red onion (whole, peel, and pulp), and yellow onion (whole, peel, and pulp).

Regarding vitamin composition, vitamin C was the most abundant compared to vitamins A and E, predominantly found in the peel, with significantly lower concentrations in whole onions and the pulp. Red onions, particularly Tropea, contained the highest levels of vitamin C. A similar distribution was observed for vitamin A, whereas vitamin E was present in much lower quantities across all three varieties, not exceeding 1 mg per 100 g in any sample (Figure 2). The assessment of antioxidant capacity, based on ABTS, DPPH, and TAC assays, confirmed that the peel exhibited the highest antioxidant activity, nearly twice that of the pulp and whole onion (Figure 3). This suggests that onion peels contain a high concentration of bioactive compounds contributing to their antioxidant properties. Whole onions displayed intermediate antioxidant activity, while the pulp had the lowest values.

In terms of mineral composition, cations and anions were more concentrated in the pulp and whole onion compared to the peel. Among cations, calcium and potassium were the most prevalent, with Tropea whole onions and pulp showing the highest levels (Table 1). Regarding anions, phosphate, sulfate, and chloride were found in greater quantities in red onions, particularly Tropea, with higher concentrations in the whole onion and pulp compared to the peels. Sulfate levels were especially pronounced in Tropea, indicating a distinct mineral profile (Table 2).

Overall, these findings highlight significant compositional differences among the three onion varieties and their respective parts. The results emphasize the nutritional and bioactive richness of the peel, particularly in proteins, phenols, flavonoids, vitamins, and antioxidants, underscoring its potential as a valuable byproduct for functional food applications. The red onion varieties, especially Tropea, consistently exhibited higher nutrient densities than the yellow onion, reinforcing their superior nutritional and functional properties.

Polyphenolic acids were more abundant in peels than in pulp and whole onions across all three varieties, with Tropea containing the highest levels of single phenolic acids. Ferulic acid (51.3 mg/100 g) was the most prevalent, followed by chlorogenic (32.8 mg/100 g), 2,5-dihydroxybenzoic, syringic (13.2 mg/100 g), protocatechuic (7.3 mg/100 g), and gallic acid (4.6 mg/100 g). These acids followed the same ranking trend in decreasing amounts in whole onions and pulp (Table 3).

Flavonoids were more abundant than phenolic acids in all three onion varieties. Spiraeoside was the most prevalent flavonoid in the peel of all cultivars, followed by quercetin, catechin, isorhamnetin-3-glucoside, vicenin-2, and cyanidin. This ranking was maintained in the pulp and whole onions, though in lower amounts. Among the cultivars, the red onion and Tropea contained the highest flavonoid levels (Table 4).

The correlation matrix analysis highlighted significant relationships among key cations, anions, and bioactive compounds in the different onion varieties examined. A strong positive correlation was found between sodium, potassium, and magnesium, suggesting that the metabolism of these elements is tightly regulated within the plant. Additionally, chlorides, nitrates, and phosphates were positively associated with cations, while fluorides and nitrites showed negative correlations (Table 5). This may indicate potential competitive interactions affecting the absorption or distribution of these ions.

In terms of variety-specific characteristics, Tropea red onion stood out with the highest concentrations of potassium and sodium, which were strongly correlated with total carbohydrate content (TCARB). This variety’s peel contained notably high levels of polyphenolic acids, enhancing its antioxidant activity, as evidenced by a strong correlation with DPPH% and total antioxidant capacity (TAC). Vitamin C levels were also notably higher, especially in the peel, highlighting the variety’s robust oxidative stress defense mechanisms. Red onion, in comparison, exhibited a more balanced distribution of minerals and bioactive compounds. While it had slightly lower polyphenol content than Tropea red onion, it still maintained a significant level of antioxidant activity. A clear correlation between flavonoids and antioxidant capacity was observed, particularly in the peel, which contained substantial amounts of quercetin and kaempferol. Yellow onion, on the other hand, was characterized by lower concentrations of polyphenolic acids and flavonoids, resulting in a comparatively reduced antioxidant profile. However, it showed higher accumulation of calcium and phosphates, suggesting that these elements might contribute more to structural stability than to metabolic activity. Additionally, vitamin E was slightly more prominent in this variety, potentially compensating for the lower flavonoid content and helping to maintain its oxidative stress response (Table 5).

**Figure 4:**
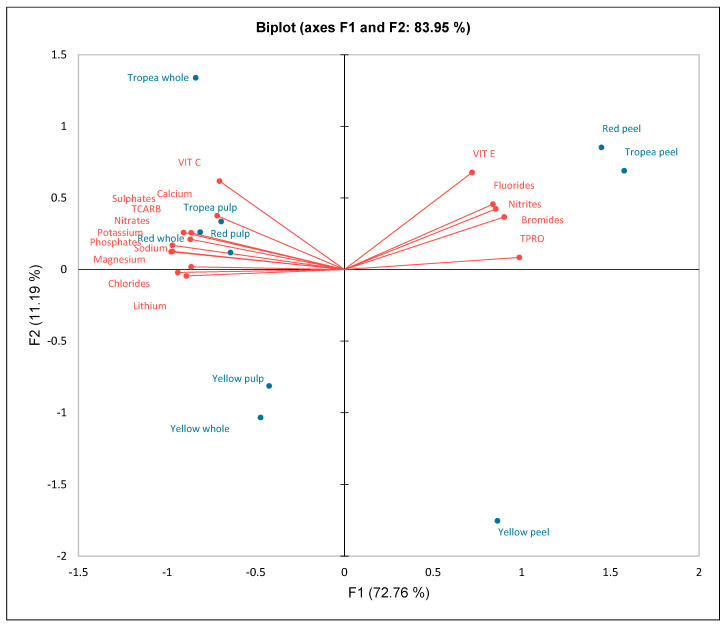
PCA of cations, anions, TCARB, TPRO, VIT A, VIT C, and VITC for Tropea red onion (whole, peel, and pulp), red onion (whole, peel, and pulp), and yellow onion (whole, peel, and pulp).

The correlation analysis further revealed that TCARB were positively correlated with potassium and sodium, indicating a connection between energy regulation and the presence of these minerals. Total proteins, however, were negatively correlated with the majority of cations, reflecting a metabolic balance between protein accumulation and ion availability. Vitamins exhibited varying trends: while vitamin C was positively correlated with potassium and sodium, vitamin E showed a strong negative correlation with fluorides and nitrites. This suggests that vitamin E may interact with the plant’s oxidative stress mechanisms in a way that modulates the effects of these compounds. A closer examination of polyphenolic acids revealed that compounds such as p-coumaric, m-coumaric, and o-coumaric exhibited extremely high correlations, indicating a common metabolic synthesis. Total antioxidant capacity was strongly associated with acids such as chlorogenic and ferulic, reinforcing their pivotal role in the onion’s antioxidant potential. On the other hand, ellagic acid showed weak or negative correlations with antioxidant activity, which may imply lower bioavailability or a different role in the plant’s physiological processes. Principal component analysis (PCA) further clarified the relationships among the various elements and bioactive compounds, visualizing distinct groups based on their behaviors. A clear separation between onion varieties emerged, with specific minerals and vitamins playing a significant role in this differentiation. Anions, particularly nitrates and phosphates, were crucial in distinguishing the samples, while the distribution of polyphenolic acids mirrored their contribution to the overall antioxidant activity (Figure 4).

## 3. Discussion

Recent research consistently indicated that plant-based dietary patterns were associated with a lower incidence of chronic diseases such as cancer, cardiovascular disease, stroke, Alzheimer’s disease, and age-related macular degeneration [15,16]. Recent research showed that, in the highly industrialized nations, these significantly contribute to burden on healthcare systems, reducing overall life expectancy [17]. The protective effects of fruits and vegetables are attributed to their high content of essential vitamins, minerals, fiber, and bioactive compounds such as polyphenols, flavonoids, and carotenoids, which possess antioxidant, anti-inflammatory, and immune-boosting properties [18,19]. Functional foods—those containing biologically active components with health-enhancing properties—have gained attention for their role in disease prevention. These foods include berries, nuts, whole grains, cruciferous vegetables, and members of the Allium family, which have been shown to modulate metabolic pathways, reduce oxidative stress, and lower inflammation [20,21]. The Allium family, which includes onions, garlic, leeks, and shallots, has been extensively studied for its health benefits. These vegetables contain a great amount of non-sulfur compounds like phenolic acids and flavonoids that exhibit strong antioxidant and anti-inflammatory properties that can change with territoriality and variety.

The nutritional analysis of Tropea, red, and yellow onions revealed significant differences in macronutrient composition, phenolic compounds, flavonoids, vitamins, antioxidant capacity, and mineral content. These variations are crucial in understanding their nutritional and functional potential, particularly for human health and food applications. All onion varieties contain substantial flavonoid concentrations, with quercetin and kaempferol derivatives being the most abundant forms [22]. These flavonoids, primarily found as glucose conjugates, contribute to onions’ strong antioxidant properties [23].

Red onions exhibited the highest flavonoid content due to the presence of anthocyanins, which enhance their antioxidant capacity and provide their distinct pigmentation [24]. The combination of anthocyanins and quercetin glycosides not only contributes to red onions’ vibrant color but also enhances their bioactivity, supporting anti-inflammatory, cardioprotective, and anticancer effects [25]. Additionally, red onions contain a greater diversity of flavonoid compounds compared to white and yellow onions, making them an excellent dietary source of polyphenols with significant health benefits [26]. Among the varieties studied, Tropea and red onions demonstrated superior nutritional value, particularly in terms of protein, carbohydrates, and bioactive compounds, compared to yellow onions.

The significantly higher protein content in Tropea and red onions suggests they may contribute more to dietary protein intake than yellow onions, consistent with previous studies linking higher protein levels in red onions to genetic and environmental factors [27]. Carbohydrate distribution analysis indicated that, while the peel is the most nutrient-dense part, the pulp remains a crucial source of energy-rich compounds. The highest total phenolic content in Tropea aligns with findings that red onions are richer in anthocyanins, quercetin, and other flavonoids, enhancing their antioxidant properties [28]. These findings support the role of Tropea red onions in cardiovascular health and anti-inflammatory effects [29].

The high levels of specific polyphenolic acids in Tropea onions suggest a strong protective role against oxidative damage, with potential anticarcinogenic, antidiabetic, and neuroprotective properties, making them a superior choice for functional food applications [30]. Their vitamin composition further highlights their functional value, with significantly higher vitamin C levels in the pulp and whole bulb, reinforcing its role in immune function, collagen synthesis, and free radical scavenging [31]. Although present in smaller amounts, vitamins A and E contribute to cellular aging prevention and vision support [32]. The limited vitamin E content across all varieties suggests onions are not a primary source of this vitamin, but their combination with other antioxidants may enhance its bioavailability. The antioxidant capacity, assessed through ABTS, DPPH, and TAC assays, was significantly high in Tropea in particular in the peel, indicating that the outer layers of onions contain concentrated bioactive compounds that can scavenge free radicals more effectively than the inner edible portions [33]. The nearly twofold difference in antioxidant activity between the peel and pulp underscores the potential of onion peels as a valuable food byproduct for developing nutraceuticals or antioxidant-rich extracts. Given the increasing interest in natural antioxidants for preventing oxidative-stress-related diseases, the high antioxidant activity of Tropea onion makes this cultivar particularly relevant for inclusion in functional foods or dietary supplements aimed at reducing the risk of chronic diseases such as cardiovascular disorders, neurodegenerative conditions, and cancer [34]. The distribution of cations and anions showed that Tropea onion contains the highest levels of essential minerals, particularly calcium and potassium. These minerals play crucial roles in bone health, muscle function, and cardiovascular regulation [35]. The higher phosphate and sulfate concentrations in red onions, particularly in Tropea, further support their enhanced nutritional profile, as these minerals are involved in metabolic processes such as energy production and detoxification pathways. Interestingly, the high sulfate content in Tropea aligns with previous research indicating that sulfate is correlated with high levels of sulfur-containing compounds, such as allyl sulfides, that have potential anticancer properties [36]. This suggests that the mineral composition of onions is not only relevant for nutritional purposes but also influences their functional bioactivity. The findings emphasize also the intricate interplay between minerals and bioactive compounds across different onion varieties. The high correlation between major cations suggests that the transport and accumulation of these elements are tightly regulated, likely through mechanisms involved in ion balance and cellular homeostasis. The relationship between minerals and secondary metabolites provides further insights. Vitamins, particularly vitamin C, appear to be closely linked to the presence of essential minerals, highlighting their role in the plant’s antioxidant defense processes. It is interesting to note from the results of the correlation matrix how phenolic acids, which are all more expressed in the Tropea variety, have different correlations with antioxidant activities. Surprisingly, meta-, ortho-, and para-coumaric acids are positively correlated with DPPH, TAC, and ABTS activities, as are trans-cinnamic, 3-hydroxycinnamic, ferulic, and chlorogenic acids, thus demonstrating a great antioxidant potential for this variety. Other phenolic acids, such as gallic, syringic, ellagic, sinapic, and caffeic acids, which are not correlated with specific antioxidant activities, nonetheless play a well-documented role at the metabolic level [37,38]. Syringic acid has a well-known effect on diabetes and cancer prevention, sinapic acid has anticonvulsant effects and reduces hippocampal neuronal damage by activating the glutamatergic pathway [39], and ellagic acid has effects on breast cancer [40]. These well-documented effects demonstrate the potential of Tropea onion [41] as a metabolic bioregulator capable of synergistically and simultaneously modulating different metabolic pathways through the action of various bioactive compounds. An additional and complementary role is played by flavonoids, most of which are correlated with antioxidant activities [42], except for rutin, cyanidin-3-glucoside, quercetin-3-glucoside, delphinidin, and procyanidin-2. However, like phenolic acids, these compounds also have important metabolic activities. In particular, cyanidin-3-glucoside has an effect on reducing metabolic disorders, quercetin-3-glucoside has an antiproliferative role on cancer cells, and delphinidin acts on stress-related genes. A review discussed their positive effects also on chronic skin conditions, including vitiligo, psoriasis, acne, and atopic dermatitis. The beneficial impacts are attributed to their antioxidant, anti-inflammatory, antimutagenic, and anticarcinogenic properties [43]. The PCA analysis clarified also the distribution of minerals and metabolites across onion varieties.

## 4. Materials and Methods

### 4.1. Sample Extract Preparation

Yellow, red, and Tropea red onions were sourced from the most common local cultivars in the Calabrian region (Italy). After harvesting, at the end of October when the bulbs were ready for sale, 10 bulbs from each variety were carefully selected to obtain representative and uniform samples. Analyses were performed on the edible portion (pulp), peels, and the whole onions. Onions were dried with an innovative process by Gioia succhi a Calabrian food transformation industry that used a physical spry dried innovative process (PDS) covered by the industrial secret. The optimized drying technology enables better preservation of thermosensitive compounds, reduces oxidation-related degradation, and ensures a more uniform and efficient drying process. This is particularly significant for maintaining the functional properties of bioactive compounds, which are often sensitive to heat and processing conditions. Additionally, the enhanced control over droplet formation and moisture removal contributes to improved solubility and dispersibility of the final dried product.

The extracts were obtained using the method described in Muscolo et al. [44]. Briefly, lyophilized onions were extracted at room temperature (22–25 °C) with continuous stirring for 90 min with 15 mL 95% ethanol. The samples were centrifuged (Unicen 21 RT167, Ortoalresa Inc., Madrid, Spain) at 2370× *g* (4000 rpm) for 15 min and the supernatants were filtered with 1 mm Whatman 185 filter paper (Merck, Rahway, NJ, USA), evaporated to dryness in a rota-vapour (Diagonal condenser RE 400, Stuart Equipment, ST15, Stone, UK), and resuspended in a final volume of 3.0 mL 95% ethanol.

Lyophilized onions (whole, pulp, and peels) were extracted at room temperature with continuous stirring for 60 min with 2.0 mL dH20 (Intercontinental Mod still 3/ES, Bioltecnical Service, s.n.c., Rome, Italy). The samples were then centrifuged at 590× *g* (2000 rpm) for 10 min and the supernatants were filtered with Whatman 1 filter paper and used for the determination of protein and carbohydrates.

### 4.2. Chemicals

Metaphosphoric acid, 2,2-diphenyl-1-picrylhydrazyl (DPPH), NaOH, nitro-blue tetrazolium, dichlorophenol-indophenol (DCPID),2,2′-azino-bis (3-ethylbenzothiazoline-6-sulfonic acid) di-ammonium salt (ABTS•+), 6-hydroxy-2,5,7,8-tetramethylchromane-2-carboxyl acid (Trolox), phenazine methosulphate, ethanol, gallic acid, ethylene-diamine-tetra acetic acid (EDTA), ferrozine, 2,4,6-tris(2-piridil)-s-triazine (TPTZ), and iron sulfate heptahydrate were purchased from Sigma Chemical Co. (St. Louis, MO, USA). HPLC-grade methanol and acetonitrile (Sigma Aldrich, St. Louis, MI, USA, 99.99%), acetone (Sigma Aldrich, 99.5%), deionized water, formic acid (Carlo Erba, 95%), and hydrochloric acid (Carlo Erba, 37%) were used for sample extraction and HPLC analysis. All chemical standards, gallic acid, protocatechuic acid, procyanidin 1 and 2, syringic acid, *p*,*m*,*o*-coumaric acids, pelargonidin, trans-cinnamic acid, bergamottin, cyanidine-3-*O*-glucoside, catechin, vanillic acid, epicatechin, delphinidin, trans-4-hydroxycinamic acid, sinapinic acid, 3-hydroxycinnamic acid, myricetin, luteolin, punicalagin, 2,5 dihydroxy benzoic acid, caffeic acid, ellagic acid, naringin, apigenin-7-neohesperosside, spiraeoside, quercetin, kaempferol, tocopherol, chlorogenic acid, vicenin 2, eriocitrine, rutin, vitexin, quercitin-3 beta-D glucoside, ferulic acid, and apigenin, were purchased from Sigma Aldrich Milano, Italy. Other chemicals were of analytical grade purchased from Carlo Erba Reagents s.r.l. (Cornaredo, Milano, Italy).

### 4.3. Protein and Carbohydrate Detection

Soluble protein was determined using the Bradford method [45] by using Coomassie Brilliant Blue G-250. The absorbance of each sample was measured at 595 nm using an 1800 UV-Vis Spectrophotometer (Shimadzu, Kyoto, Japan). Bovine serum albumin >99% purity (Sigma) was used as standard, and soluble proteins were estimated as mg BSA/g dw.

The total available carbohydrates were measured using the anthrone method with minor modifications as reported in Muscolo et al. [46]. The amount of available carbohydrates was calculated using a glucose calibration curve (range of 10–100 mg/mL). The results were reported as mg/g dw.

### 4.4. Determination of Total Phenolic Compounds, Total Flavonoids, and Vitamins A, C, and E

Total phenol content was determined with the Folin–Ciocalteu reagent according to Muscolo et al. [46]. Briefly, 500 µL of the aqueous extract was mixed with 250 µL of Folin–Ciocalteu reagent and 2 mL of a 20% Na_2_ CO_3_ aqueous solution; the mixture was filled up to 50 mL with deionized water and placed in the dark for 1 h. The absorbance was measured at 765 nm using a UV–Vis Agilent 8453 spectrophotometer (Agilent Technologies, CA, USA). The results were expressed as mg/L of gallic acid equivalents.

Total flavonoid content was determined according to the colorimetric method as reported in Muscolo et al. [46]. The absorbance was measured at 510 nm using a UV–Vis Agilent 8453 spectrophotometer (Agilent Technologies, Santa Clara, CA, USA). The results were expressed as rutin equivalents (mg/L) using a calibration curve.

Vitamin A was detected as reported in Aremu and Nweze [47]. Absorbance was read at 436 nm and vitamin A was expressed as Retinol Equivalent (RE).

For vitamin C (ascorbic acid) determination, the method of Davies and Masten [48] was used. Pomegranate powders (0.10 g) were extracted with 10 mL of 3% meta-phosphoric acid—7.98% acetic acid was centrifuged at 2370× *g* (4000 rpm) for 10 min and the supernatant was used for the determination of ascorbic acid.

For vitamin E (α-tocopherol) analysis, pomegranate powder (0.10 g) was extracted with 10 mL of hexane/isopropanol solution (3:2 *v*/*v*), with agitation for 5 h, and centrifuged at 1330× *g* (3000 rpm) for 10 min. The supernatant was used for the determination of vitamin E [49].

### 4.5. Determination of Antioxidant Activities

The antioxidant activity against DPPH radical (2,2-diphenyl-1-picryl-hydrazyl-hydrate) was determined with the method reported in Muscolo et al. [46]. The DPPH concentration in the cuvette was chosen to give absorbance values of ∼1.0. Absorbance changes of the violet solution were recorded at 517 nm after 30 min of incubation at 37 °C. The inhibition I (%) of radical-scavenging activity was calculated as:

I (%) = [(A0 − AS)/A0] × 100,

where A0 is the absorbance of the control and AS is the absorbance of the sample after 30 min of incubation. Results were expressed as µmol Trolox/g DW.

The 2,2′-azino-bis-3-ethylbenzothiazoline-6-sulfonic acid assay (ABTS) was carried out according to Muscolo et al. [46] by using a solution of 7 mM of ABTS in phosphate buffered saline (PBS). Aliquots of ethanol extracts (25, 50, and 100 μL) were added to 0.5 mL of ABTS+• solution and brought to a final volume of 600 μL with PBS. After 6 min of incubation in the dark at room temperature, the absorbance of the samples was measured at 734 nm. Results were expressed as µmol Trolox/g DW.

The total antioxidant capacity (TAC) was performed according to Muscolo et al. [46]. Sample absorbance was measured at 695 nm using UV–visible spectrophotometer. Methanol (0.3 mL) in place of extract was used as a blank. The antioxidant activity was expressed as μg of α-tocopherol g^−1^ DW on a calibration curve.

### 4.6. RP-DAD-HPLC Identification of Phenolic and Flavonoid Components

Dried onion and its parts were subjected to solvent extraction before HPLC–PDA/ESI-MS analysis for determination of the single phenolic and flavonoid compounds. Each sample was extracted in two different ways: 0.1 g of previously lyophilized pomegranate peels was dissolved in 10 mL of 1% of HCl in methanol and 0.1 g of sample was dissolved in 10 mL of acetone solution/1% of HCl in methanol (1:1). Each sample was analyzed in three independent replicates [46]. A panel of analytical standards was used for flavonoid and phenolic profiling. The following HPLC-grade standards were used: gallic acid, protocatechuic acid, *p*-hydroxybenzoic acid, catechin, chlorogenic acid, caffeic acid, syringic acid, epicatechin, *p*-*m*-*o*-coumaric acid, epicatechin gallate, ferulic acid, quercetin-3-*O*-glucuronide, quercetin-3-*O*-galactoside, kaempferol-3-*O*-glucoside, quercetin, kaempferol, acetic acid, and acetonitrile. All standards and solvents were of analytical grade and suitable for chromatographic analysis. HPLC vials (1.5 mL) were supplied by Agilent Technologies (Mulgrave, VIC, Australia).

### 4.7. Statistical Analysis

Analysis of variance (ANOVA) was performed on all data sets. One-way ANOVA, followed by Tukey’s Honestly Significant Difference (HSD) test, was used to assess the effects of treatment and cultivar on each of the parameters measured. Both ANOVA and *t*-tests were conducted using SPSS software (IBM Corp., 2012). Statistical significance was determined at *p* ≤ 0.05. To explore relationships among different treatments, cultivars, and chemical parameters, Principal Component Analysis (PCA) was applied to the data sets.

For PCA purposes, missing values indicated as “n.d.” were replaced using a conservative and standardized approach. Additionally, a correlation matrix was generated to explore the linear relationships between minerals, macronutrients, and vitamins. The matrix was calculated using Pearson’s correlation coefficient (r) in XLSTAT software (Addinsoft, Paris, France) and visualized using a color scale to indicate the strength and direction of correlations.

## 5. Conclusions

In conclusion, while consuming vegetables is essential for a healthy diet, the concentration of bioactive compounds within them is often too low to achieve significant therapeutic effects. Extracting bioactive compounds from vegetables is crucial for developing natural health integrators that help prevent diseases. These compounds, such as polyphenols, flavonoids, and vitamins, possess antioxidant, anti-inflammatory, and immune-boosting properties. By incorporating them into supplements, they can enhance overall well-being, reducing the risk of chronic conditions like cardiovascular diseases and cancer and support a healthier lifestyle. Utilizing vegetable-derived bioactive compounds also promotes sustainable solutions for preventive healthcare. Creating integrators allows for a controlled and optimized intake of these beneficial compounds, ensuring the body receives an effective dose to support health and disease prevention. Formulated supplements provide a standardized amount of key bio-actives, such as polyphenols, flavonoids, and vitamins, which would otherwise require consuming impractically large quantities of vegetables. This targeted approach enhances bioavailability, maximizing the preventive and therapeutic benefits of these natural compounds. Based on the comprehensive analysis of macronutrients, bioactive compounds, antioxidant potential, and mineral composition. The observed differences suggest that both genetic and environmental factors significantly influence the chemical composition of onions, with important implications for human nutrition and agro-food applications. Notably, the importance of nitrates and phosphates in differentiating varieties suggests their potential as markers of metabolic variation. Tropea onion emerges as the most nutritionally valuable variety. Its high protein, phenolic, flavonoid, and vitamin C content, along with superior antioxidant activity, makes it an ideal choice for potential functional food applications and it can be eligible for the production of metabolic bioactivators.

## Figures and Tables

**Table 1:** Cations (mg/g) in Tropea red onion (whole, peel, and pulp), red onion (whole, peel, and pulp), and yellow onion (whole, peel, and pulp). Data are the mean of three replicates (*n* = 18) ± standard deviation. Different letters in the same column indicate statistically significant differences (Tukey’s test, *p* ≤ 0.05) between the means of the tested groups. If two values share the same letter, it means they are not significantly different, whereas different letters denote a significant difference between groups.

Cations	Lithium	Sodium	Potassium	Magnesium	Calcium
Tropea peel	n.d.	0.5 ± 0.02	1.1 ^c^ ± 0.04	0.2 ^c^ ± 0.01	7.7 ^c^ ± 0.02
Red peel	n.d.	0.7 ^d^ ± 0.05	1.1 ^c^ ± 0.01	0.2 ^c^ ± 0.21	7.6 ^c^ ± 0.12
Yellow peel	n.d.	0.5 ^d^ ± 0.01	1.1 ^c^ ± 0.12	0.2 ^c^ ± 0.08	7.6 ^c^ ± 0.23
Tropea whole	0.04 ^a^ ± 0.01	1.44 ^c^ ± 0.32	39.8 ^a^ ± 1.56	2.8 ^a^ ± 0.04	16.1 ^a^ ± 0.11
Red whole	0.09 ^a^ ± 0.01	1.80 ^b^ ± 0.01	35.8 ^b^ ± 2.01	2.4 ^a^ ± 0.01	8.5 ^c^ ± 0.32
Yellow whole	0.06 ^a^ ± 0.01	1.64 ^b^ ± 0.01	34.2 ^c^ ± 2.45	2.3 ^a^ ± 0.02	10.1 ^b^ ± 0.12
Tropea pulp	0.07 ^a^ ± 0.01	2.31 ^a^ ± 0.01	38.4 ^a^ ± 3.01	2.7 ^a^ ± 0.07	10.5 ^b^ ± 0.23
Red pulp	0.07 ^a^ ± 0.01	2.63 ^a^ ± 0.01	31.9 ^b^ ± 1.01	2.6 ^a^ ± 0.02	12.5 ^b^ ± 0.16
Yellow pulp	0.07 ^a^ ± 0.01	2.28 ^a^ ± 0.01	31.6 ^b^ ± 4.01	2.5 ^a^ ± 0.04	11 ^b^ ± 0.19

**Table 2:** Anions (mg/g) in Tropea red onion (whole, peel, and pulp), red onion (whole, peel, and pulp), and yellow onion (whole, peel, and pulp). Data are the mean of three replicates (*n* = 18) ± standard deviation. Different letters in the same column indicate statistically significant differences (Tukey’s test, *p* ≤ 0.05) between the means of the tested groups. If two values share the same letter, it means they are not significantly different, whereas different letters denote a significant difference between groups.

Anions	Fluorides	Chlorides	Nitrites	Bromides	Nitrates	Phosphates	Sulfates
Tropea peel	0.011 ^a^ ± 0.03	0.123 ^b^ ± 0.05	0.029 ^a^ ± 0.01	0.044 ^a^ ± 0.01	0.004 ^c^ ± 0.01	n.d.	n.d.
Red peel	0.010 ^a^ ± 0.06	0.122 ^b^ ± 0.04	0.032 ^a^ ± 0.03	0.039 ^a^ ± 0.03	n.d.	n.d.	0.055 ^c^ ± 0.01
Yellow peel	0.002 ^a^ ± 0.01	0.153 ^d^ ± 0.03	n.d.	0.008 ^b^ ± 0.08	n.d.	n.d.	0.072 ^c^ ± 0.01
Tropea whole	0.003 ^a^ ± 0.01	0.345 ^b^ ± 0.09	n.d.	0.001 ^c^ ± 0.01	0.032 ^a^ ± 0.01	0.827 ^a^ ± 0.08	0.193 ^a^ ± 0.02
Red whole	0.002 ^a^ ± 0.03	0.306 ^b^ ± 0.07	n.d.	0.008 ^b^ ± 0.02	0.026 ^a^ ± 0.01	0.831 ^a^ ± 0.02	0.139 ^b^ ± 0.05
Yellow whole	0.003 ^a^ ± 0.07	0.408 ^a^ ± 0.02	0.002 ^b^ ± 0.02	0.001 ^c^ ± 0.01	0.025 ^a^ ± 0.02	0.596 ^c^ ± 0.06	0.096 ^c^ ± 0.08
Tropea pulp	0.002 ^a^ ± 0.01	0.345 ^b^ ± 0.04	n.d.	0.001 ^c^ ± 0.02	0.012 ^b^ ± 0.01	0.859 ^a^ ± 0.01	0.187 ^a^ ± 0.02
Red pulp	0.002 ^a^ ± 0.01	0.410 ^a^ ± 0.01	n.d.	n.d.	0.027 ^a^ ± 0.01	0.953 ^a^ ± 0.23	0.150 ^b^ ± 0.01
Yellow pulp	0.002 ^a^ ± 0.06	0.295 ^c^ ± 0.12	n.d.	n.d.	0.016 ^b^ ± 0.05	0.706 ^b^ ± 0.12	0.083 ^c^ ± 0.03

**Table 3:** Polyphenolic acids (mg/g) in Tropea red onion (whole, peel, and pulp), red onion (whole, peel, and pulp), and yellow onion (whole, peel, and pulp). Different letters in the same row indicate statistically significant differences (Tukey’s test, *p* ≤ 0.05) between the means of the tested groups. If two values share the same letter, it means they are not significantly different, whereas different letters denote a significant difference between groups.

	PEEL			PULP			WHOLE		
Polyphenolic Acids	Tropea	Red	Yellow	Tropea	Red	Yellow	Tropea	Red	Yellow
	mg/100 g	mg/g	mg/g	mg/g	mg/g	mg/g	mg/g	mg/g	mg/g
Gallic acid	4.6 ± 0.7 ^a^	0.87 ± 0.7 ^c^	0.4 ± 0.04 ^c^	0.33 ± 0.07 ^c^	0.2 ± 0.05 ^c^	1.6 ± 0.5 ^b^	3.4 ± 0.5 ^ab^	0.8 ± 0.03 ^c^	0.53 ± 0.03 ^c^
Protocathecuic acid	7.3 ± 2 ^a^	10.3 ± 1.9 ^a^	n.d.	1.4 ± 0.3 ^d^	0.8 ± 0.3 ^d^	0.6 ± 0.05 ^d^	4.8 ± 0.9 ^b^	1.8 ± 0.07 ^c^	1.93 ± 0.06 ^c^
Siryngic acid	13.2 ± 1.1 ^a^	4.2 ± 0.6 ^b^	n.d.	n.d.	n.d.	2.5 ± 0.23 ^c^	n.d.	n.d.	n.d.
*p*-Coumaric acid	4.8 ± 0.3 ^a^	4.6 ± 0.2 ^a^	3.3 ± 0.5 ^ab^	1.47 ± 0.9 ^c^	1.5 ± 0.8 ^c^	2.23 ± 0.6 ^b^	2.31 ± 0.6 ^b^	2.5 ± 0.7 ^b^	2.5 ± 0.6 ^b^
*m*-Coumaric acid	6.8 ± 1.3 ^a^	2.5 ± 0.5 ^b^	0.6 ± 0.02 ^c^	0.3 ± 0.02 ^c^	0.3 ± 0.04 ^c^	0.3 ± 0.02 ^c^	0.30 ± 0.2 ^c^	0.4 ± o.o3 ^c^	0.3 ± 0.01 ^c^
*o*-Coumaric acid	6.9 ± 1.3 ^a^	8.7 ± 1.2 ^a^	0.97 ± 0.6 a^bc^	0.36 ± 0.03 ^c^	1.5 ± 0.6 ^b^	0.4 ± 0.06 ^c^	1.5 ± 0.5 ^b^	1.5 ± 0.3 ^b^	1.5 ± 0.6 ^b^
*Trans*-cinnamic acid	3.6 ± 0.6 ^a^	3.3 ± 0.4 ^a^	1.4 ± 0.5 ^b^	0.6 ± 0.03 ^c^	0.64 ± 0.05 ^c^	0.65 ± 0.03 ^c^	0.65 ± 0.04 ^c^	0.62 ± 0.06 ^c^	0.63 ± 0.02 ^c^
Vanillic acid	1 ± 0.5 ^a^	1.5 ± 0.9 ^a^	0.4 ± 0.03 ^c^	0.6 ± 0.03 ^b^	0.1 ± 0.01 ^c^	0.3 ± 0.03 ^c^	0.23 ± 0.01 ^c^	0.33 ± 0.02 ^c^	0.33 ± 0.03 ^c^
*Trans*-4-hydroxycinnamic acid	0.23 ± 0.03 ^c^	0.23 ± 0.06 ^c^	0.2 ± 0.02 ^c^	1.33 ± 0.6 ^a^	0.13 ± 0.06 ^c^	0.22 ± 0.01 ^c^	0.55 ± 0.06 ^b^	0.133 ± 0.01 ^c^	0.142 ± 0.03 ^c^
Sinapic acid	1.7 ± 0.6 ^a^	0.4 ± 0.02 ^b^	0.2 ± 0.01 ^b^	1.3 ± 0.4 ^a^	0.27 ± 0.03 ^b^	0.35 ± 0.02 ^b^	0.40 ± 0.03 ^b^	0.33 ± 0.04 ^b^	0.30 ± 0.02 ^b^
3-hydroxycinnamic acid	3 ± 0.5 ^a^	2.2 ± 0.1 ^b^	0.2 ± 0.01 ^c^	0.1 ± 0.02 ^c^	0.1 ± 0.02 ^c^	n.d.	0.1 ± 0.02 ^c^	n.d.	0.2 ± 0.02 ^c^
2,5-dihydroxybenzoic acid	16.8 ± 2 ^a^	5.5 ± 1.1 ^bc^	3.8 ± 0.9 ^c^	9.1 ± 1.2 ^b^	0.5 ± 0.02 ^d^	3.3 ± 0.8 ^b^	9.2 ± 1.4 ^b^	2.2 ± 0.7 ^c^	2.4 ± 0.8 ^c^
Caffeic acid	4.4 ± 1.0 ^b^	3.8 ± 0.9 ^b^	0.67 ± 0.02 ^c^	0.25 ± 0.02 ^c^	0.2 ± 0.02 ^c^	0.1 ± 0.01 ^c^	9.27 ± 1.2 ^a^	0.13 ± 0.02 ^c^	0.1 ± 0.01 ^c^
Ellagic acid	6 ± 1.1 ^b^	1.36 ± 0.2 ^bc^	18.7 ± 2.2 ^a^	1.1 ± 0.2 ^bc^	0.47 ± 0.02 ^c^	0.1 ± 0.01 ^c^	0.43 ± 0.03 ^c^	0.57 ± 0.04 ^c^	2.8 ± 0.5 ^b^
Chlorogenic acid	32.8 ± 1.9 ^a^	18.9 ± 1.7 ^b^	3.6 ± 0.5 ^d^	4.1 ± 0.5 ^d^	0.83 ± 0.02 ^g^	2.8 ± 0.3 ^f^	8.5 ± 1 ^c^	1.7 ± 0.4 ^e^	0.87 ± 0.03 ^g^
Ferulic acid	51.3 ± 1.6 ^a^	51.1 ± 1.2 ^a^	50.1 ± 1.7 ^a^	25.6 ± 1.6 ^b^	25.3 ± 1.8 ^b^	48.1 ± 2 ^a^	25.3 ± 1.9 ^b^	25.3 ± 1.6 ^b^	25.4 ± 1.7 ^b^

**Table 4:** Flavonoids (flavonols, flavons, and flavanonons (mg/100 g) and anthocyanins (mg/100 g) in Tropea red onion (whole, peel, and pulp), red onion (whole, peel, and pulp), and yellow onion (whole, peel, and pulp). Different letters in the same row indicate statistically significant differences (Tukey’s test, *p* ≤ 0.05) between the means of the tested groups. If two values share the same letter, it means they are not significantly different, whereas different letters denote a significant difference between groups.

	PEEL			PULP			WHOLE		
Flavonoids	Tropea	Red	Yellow	Tropea	Red	Yellow	Tropea	Red	Yellow
Isorhametin-3-glucoside	60 ± 2 ^a^	35 ± 2.1 ^b^	11 ± 1.2 ^e^	17 ± 3 ^d^	25 ± 3 ^c^	16 ± 1.2 ^d^	22 ± 2 ^c^	17 ± 2 ^d^	12 ± 1 ^e^
Isorhamnetin	19.6 ± 3 ^a^	9.5 ± 2 ^b^	7.4 ± 1.2 ^b^	1.2 ± 0.6 ^c^	1.4 ± 0.5 ^c^	1.6 ± 0.9 ^c^	0.2 ± 0.06 ^d^	0.8 ± 0.03 ^d^	1.8 ± 0.6 ^c^
Apigenin	16 ± 1.4 ^a^	12 ± 0.9 ^b^	6.8 ± 0.6 ^b^	n.d.	n.d.	n.d.	0.6 ± 0.06 ^c^	0.3 ± 0.02 ^c^	0.1 ± 0.02 ^c^
Procyanidine 2	72 ± 5.6 ^a^	86 ± 4.6 ^b^	68 ± 3.6 ^c^	4.4 ± 0.6 ^f^	4.8 ± 0.7 ^f^	4.2 ± 2.6 ^f^	42 ± 3.7 ^e^	44 ± 4.1 ^e^	54 ± 3.7 ^d^
Cyanidin-3-*O*-glucoside	29.6 ± 1.7 ^c^	19.6 ± 1.0 ^d^	10.3 ± 0.7 ^e^	112.4 ± 6.7 ^a^	128.2 ± 7 ^a^	103.2 ± 8 ^a^	115.2 ± 7 ^a^	101 ± 8 ^a^	83.8 ± 9 ^b^
Catechin	361 ± 14 ^b^	573 ± 18 ^a^	120 ± 11 ^c^	17 ± 1 ^e^	17 ± 1.2 ^e^	14 ± 1.5 ^e^	39 ± 1.5 ^d^	43 ± 1.7 ^d^	50 ± 2.1 ^d^
Myricetin	8.4 ± 1.2 ^a^	7.2 ± 1 ^a^	6.4 ± 1.1 ^a^	0.6 ± 0.05 ^c^	0.4 ± 0.04 ^c^	0.6 ± 0.06 ^c^	0.4 ± 0.03 ^c^	1.4 ± 0.3 ^b^	0.8 ± 0.07 ^c^
Luteolin	404 ± 33 ^a^	460 ± 27 ^a^	53 ± 18 ^b^	8.8 ± 10 ^c^	14 ± 1.5 ^c^	5.6 ± 1 ^d^	41.4 ± 18 ^b^	42.2 ± 16 ^b^	0.6 ± 0.06 ^e^
Spiraeoside	691 ± 32 ^a^	786 ± 28 ^b^	184 ± 14 ^c^	133 ± 18 ^d^	206 ± 15 ^c^	121 ± 11 ^d^	178 ± 15 ^b^	152 ± 18 ^b^	161 ± 14 ^b^
Quercetin	419 ± 21 ^b^	491 ± 18 ^a^	251 ± 18 ^c^	12.8 ± 1.8 ^d^	4.4 ± 0.5 ^g^	10 ± 0.7 ^f^	34 ± 36 ^e^	29 ± 1.8 ^e^	12 ± 1.6 ^f^
Kaempferol	15 ± 1.9 ^a^	19 ± 1.9 ^a^	4.4 ± 1.2 ^b^	0.2 ± 0.05 ^c^	1 ± 0.07 ^c^	n.d.	1.4 ± 0.08 ^c^	1.6 ± 0.06 ^c^	0.4 ± 0.01 ^d^
Vicenin-2	34.2 ± 18 ^a^	32.2 ± 12 ^a^	10.6 ± 5 ^b^	4.6 ± 1.2 ^c^	9.2 ± 2 ^b^	3.4 ± 1 ^d^	7.6 ± 1.3 ^b^	6.4 ± 1.2 ^b^	3.5 ± 0.8 ^d^
Rutin	0.4 ± 0.01 ^c^	3 ± 0.6 ^a^	0.8 ± 0.06 ^d^	0.6 ± 0.2 ^c^	1.2 ± 0.3 ^b^	0.4 ^c^	0.2 ± 0.02 ^c^	1.3 ± 0.2 ^b^	0.4 ± 0.01 ^c^
Quercetin-3 beta-D-glucoside	12.2 ± 1.^a^	11.4 ± 1.1 ^a^	2.8 ± 0.8 ^c^	8.4 ± 1.6 ^b^	8.2 ± 1.8 ^b^	1.8 ± 0.8 ^c^	7.4 ± 1.1 ^b^	6.5 ± 1.3 ^b^	1.1 ± 0.4 ^c^
Naringenin	1.2 ± 0.1 ^a^	1 ± 0.2 ^a^	0.6 ± 0.02 ^b^	n.d.	n.d.	n.d.	n.d.	n.d.	n.d.
Delphinidin	7.8 ± 1.4 ^a^	4.8 ± 1 ^b^	1.2 ± 0.3 ^c^	0.6 ± 0.1 ^d^	1.2 ± 0.3 ^c^	0.2 ± 0.01 ^e^	2.9 ± 1 ^bc^	2.7 ± 1.1 ^bc^	0.6 ± 0.03 ^c^
Naringin	2.2 ± 0.3 ^a^	2.7 ± 0.4 ^a^	3.2 ± 0.4 ^a^	1 ± 0.5 ^b^	0.2 ± 0.02 ^c^	1 ± 0.3 ^b^	0.6 ± 0.3 ^b^	0.8 ± 0.1 ^b^	0.6 ± 0.2 ^b^

**Table 5:** Correlation matrix (Pearson (n)) of cations, anions, TCARB, TPRO, VIT A, VIT C, and VITC in Tropea red onion (whole, peel, and pulp), red onion (whole, peel, and pulp), and yellow onion (whole, peel, and pulp).

Variables	Lithium	Sodium	Potassium	Magnesium	Calcium	Fluorides	Chlorides	Nitrites	Bromides	Nitrates	Phosphates	Sulfates	TCARB	TPRO	VIT A	VIT C	VIT E
Lithium	1	0.904	0.898	0.902	0.402	−0.712	0.838	−0.701	−0.731	0.750	0.920	0.659	0.846	−0.911	0.158	0.570	−0.653
Sodium	0.904	1	0.842	0.891	0.534	−0.671	0.851	−0.649	−0.735	0.662	0.918	0.675	0.723	−0.844	0.043	0.562	−0.599
Potassium	0.898	0.842	1	0.991	0.711	−0.714	0.921	−0.741	−0.803	0.874	0.965	0.834	0.927	−0.968	0.218	0.759	−0.594
Magnesium	0.902	0.891	0.991	1	0.742	−0.724	0.929	−0.747	−0.817	0.869	0.982	0.825	0.915	−0.966	0.163	0.734	−0.607
Calcium	0.402	0.534	0.711	0.742	1	−0.450	0.682	−0.513	−0.604	0.765	0.707	0.730	0.694	−0.653	−0.015	0.671	−0.280
Fluorides	−0.712	−0.671	−0.714	−0.724	−0.450	1	−0.722	0.988	0.970	−0.570	−0.722	−0.711	−0.662	0.836	−0.110	−0.340	0.953
Chlorides	0.838	0.851	0.921	0.929	0.682	−0.722	1	−0.745	−0.830	0.872	0.914	0.754	0.770	−0.936	0.412	0.664	−0.648
Nitrites	−0.701	−0.649	−0.741	−0.747	−0.513	0.988	−0.745	1	0.972	−0.625	−0.735	−0.713	−0.689	0.848	−0.136	−0.363	0.939
Bromides	−0.731	−0.735	−0.803	−0.817	−0.604	0.970	−0.830	0.972	1	−0.669	−0.790	−0.756	−0.701	0.900	−0.217	−0.423	0.918
Nitrates	0.750	0.662	0.874	0.869	0.765	−0.570	0.872	−0.625	−0.669	1	0.861	0.698	0.882	−0.864	0.275	0.696	−0.456
Phosphates	0.920	0.918	0.965	0.982	0.707	−0.722	0.914	−0.735	−0.790	0.861	1	0.847	0.921	−0.941	0.055	0.767	−0.582
Sulfates	0.659	0.675	0.834	0.825	0.730	−0.711	0.754	−0.713	−0.756	0.698	0.847	1	0.828	−0.799	−0.076	0.880	−0.481
TCARB	0.846	0.723	0.927	0.915	0.694	−0.662	0.770	−0.689	−0.701	0.882	0.921	0.828	1	−0.893	−0.031	0.771	−0.494
TPRO	−0.911	−0.844	−0.968	−0.966	−0.653	0.836	−0.936	0.848	0.900	−0.864	−0.941	−0.799	−0.893	1	−0.278	−0.633	0.754
VIT A	0.158	0.043	0.218	0.163	−0.015	−0.110	0.412	−0.136	−0.217	0.275	0.055	−0.076	−0.031	−0.278	1	−0.048	−0.261
VIT C	0.570	0.562	0.759	0.734	0.671	−0.340	0.664	−0.363	−0.423	0.696	0.767	0.880	0.771	−0.633	−0.048	1	−0.084
VIT E	−0.653	−0.599	−0.594	−0.607	−0.280	0.953	−0.648	0.939	0.918	−0.456	−0.582	−0.481	−0.494	0.754	−0.261	−0.084	1

## Data Availability

No new data were created or analyzed in this study. Data sharing is not applicable to this article.
